# Supplementary material for: Systemic inflammation is associated with worse outcomes from SARS-CoV-2 infection but not neutralizing antibody
Source: Microbiol Spectr. 2025 Feb 19;13(4):e02459-24. doi: 10.1128/spectrum.02459-24 (PMC11960045; doi:10.1128/spectrum.02459-24)
Supplement: Supplemental material — Fig. S1 and S2; Tables S1 and S2. [file spectrum.02459-24-s0001.pdf]

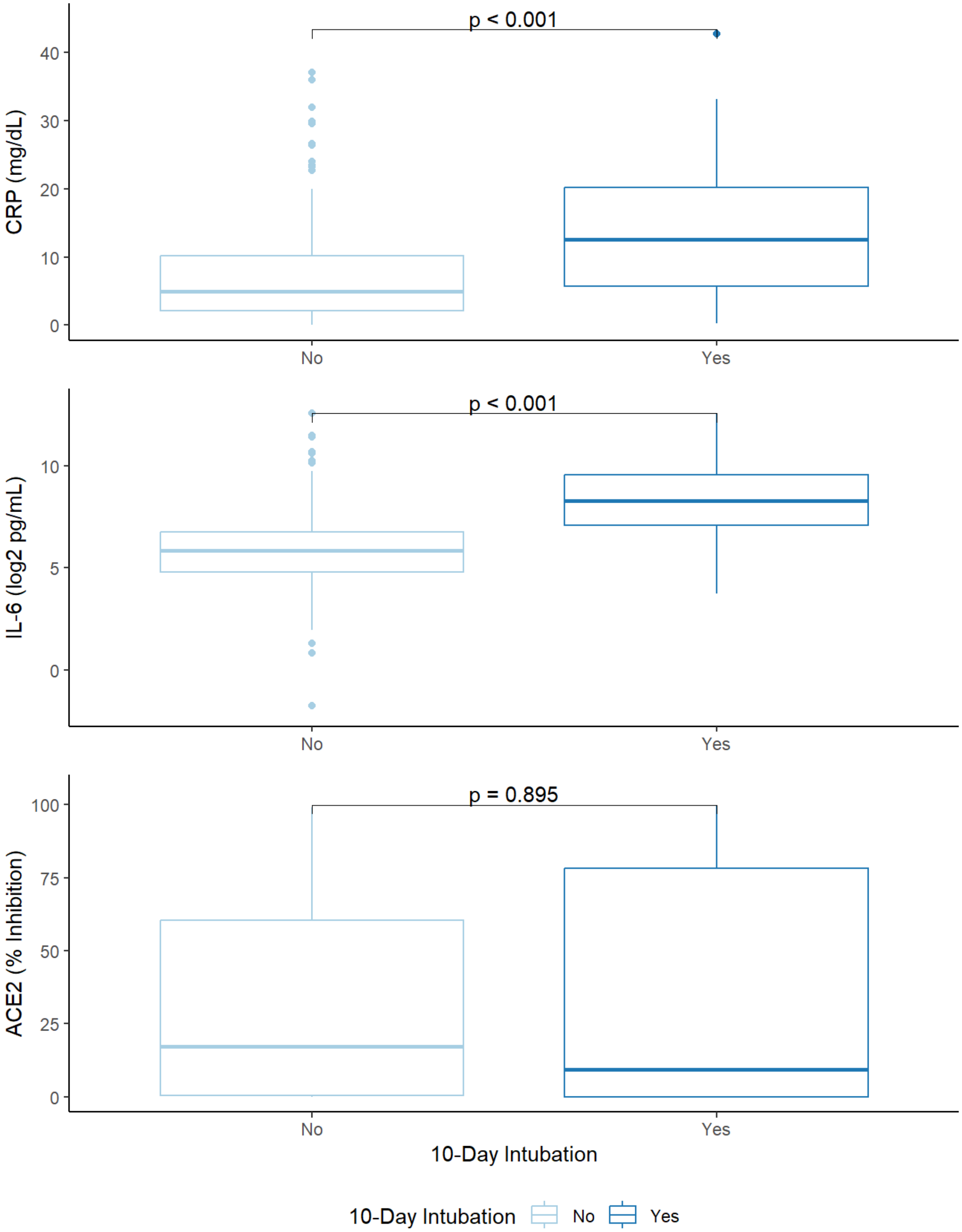

Supplemental Fig 1. Association between CRP (top), IL6 (middle) and ACE-2 Inhibition (bottom) with 10-day intubation. Lines are median, boxes represent the IQR, whiskers are the central 95%.

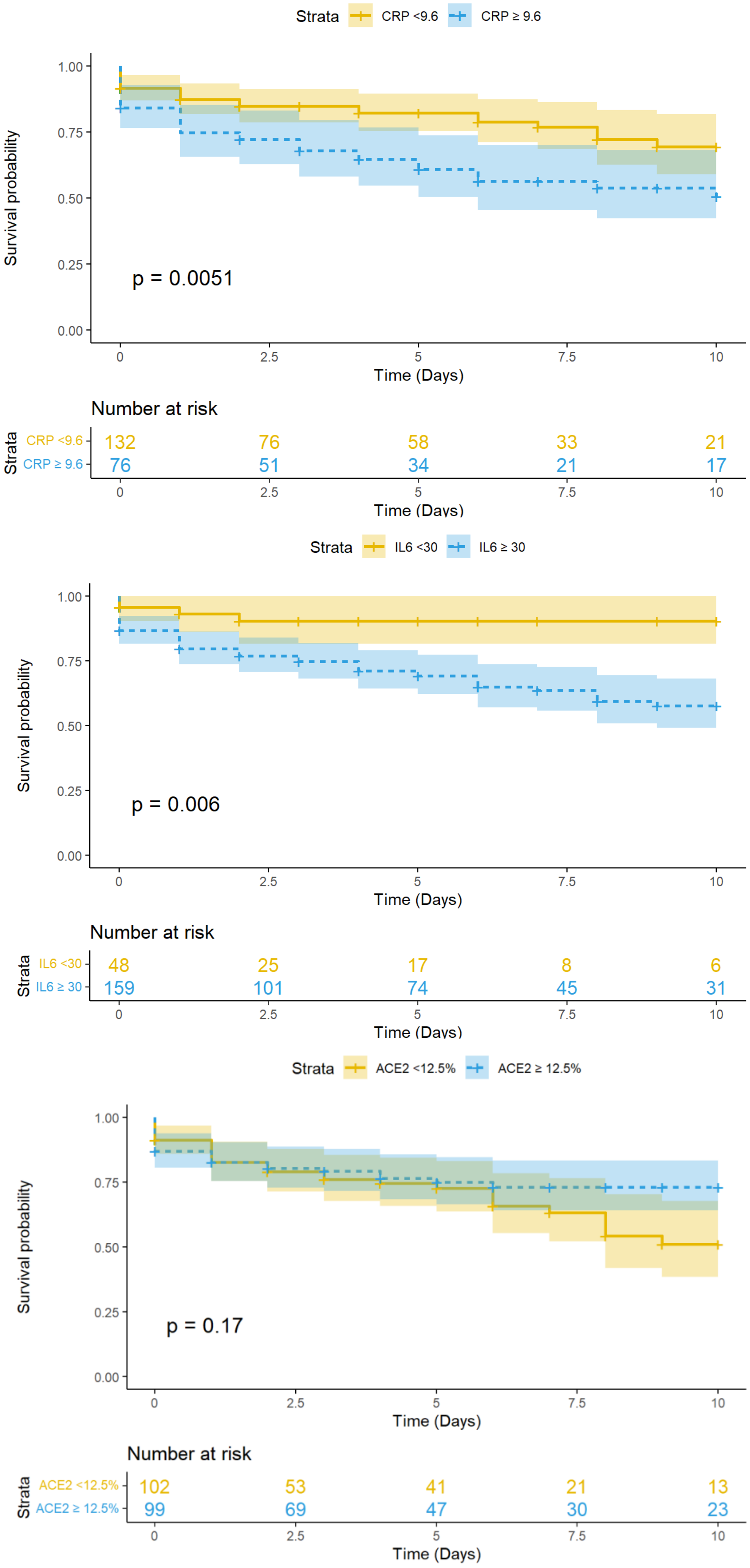

Supplemental Figure 2 Kaplan-Meier Survival curves for requirement of 10-day intubation in those below (yellow) and above (blue) designated thresholds for CRP, IL-6, and ACE-2 inhibition

Supplemental Fig 2

Supplemental Table 1 Cox proportional hazard models for 10-day intubation

Characteristic	N	HR[1]	95% CI[1]	p-value
ACE2	200	1.00	0.99, 1.01	0.7
IL-6 (log2)	200	1.47	1.29, 1.68	<0.001
CRP (log2)	200	0.99	0.84, 1.18	>0.9
Age	200	1.00	0.98, 1.02	0.9
Sex	200			
Female		—	—	
Male		0.71	0.40, 1.24	0.2
Symptom Onset (Days)	200	1.00	0.97, 1.02	0.8

[1] HR = Hazard Ratio, CI = Confidence Interval

Supplemental Table 2 Cox proportional hazard models for 30-day mortality

Characteristic	N	HR[1]	95% CI[1]	p-value
ACE2	191	0.99	0.98, 1.00	0.2
IL-6 (log2)	191	1.30	1.08, 1.57	0.006
CRP (log2)	191	1.07	0.81, 1.43	0.6
Age	191	1.05	1.02, 109	0.005
Sex	191			
Female		—	—	
Male		0.6	0.28, 1.31	0.2
Symptom Onset (Days)	191	0.96	0.91, 1.02	0.2

[1] HR = Hazard Ratio, CI = Confidence Interval, 9 patients with race undocumented in the EMR were excluded.
